# Measurement properties of the EQ-5D across four major geriatric conditions: Findings from TOPICS-MDS

**DOI:** 10.1186/s12955-017-0616-x

**Published:** 2017-03-03

**Authors:** Jennifer E. Lutomski, Paul F. M. Krabbe, Nienke Bleijenberg, Jeanett Blom, Gertrudis I. J. M. Kempen, Janet MacNeil-Vroomen, Maaike E. Muntinga, Ewout Steyerburg, Marcel G. M. Olde-Rikkert, René J. F. Melis, on behalf of TOPICS Consortium, N. Bleijenberg, J. W. Blom, G. I. J. M. Kempen, P. F. M. Krabbe, R. J. F. Melis, M. E. Muntinga, E. W. Steyerberg, B. M. Buurman, J. Gussekloo, H. E. van der Horst, M. G. M. Olde-Rikkert, S. E. J. A. de Rooij, J. M. G. A. Schols, M. J. Schuurmans, D. A Smilde, D. van den Brink, J. E. Lutomski, L. Qin

**Affiliations:** 1 Department of Geriatric Medicine, Radboud University Medical Center, Postbus 9101, Nijmegen, 6500 HB Netherlands; 2 Department of Epidemiology, University of Groningen, University Medical Center Groningen, Groningen, Netherlands; 3 Julius Center for Health Sciences and Primary Care, University Medical Center Utrecht, Utrecht, Netherlands; 4 Department of Public Health and Primary Care, Leiden University Medical Center, Leiden, Netherlands; 5 Department of Health Services Research, Care and Public Health Research Institute (CAPHRI), Maastricht University, Maastricht, Netherlands; 6 Department. of General Medicine, Academic Medical Center, Amsterdam, Netherlands; 7 Department of Internal Medicine, Yale School of Medicine, New Haven, CT USA; 8 Department of General Practice and Elderly Care Medicine/EMGO+ Institute for Health and Care Research, VU Medical Center, Amsterdam, Netherlands; 9 Department of Public Health, Erasmus MC University Medical Center, Rotterdam, Netherlands

**Keywords:** Quality of life, EQ-5D, Hearing loss, Joint diseases, Urinary incontinence, Dizziness

## Abstract

**Background:**

As populations age, chronic geriatric conditions linked to progressive organ failure jeopardize health-related quality of life (HRQoL). Thus, this research assessed the validity and applicability of the EQ-5D (a common HRQoL instrument) across four major chronic geriatric conditions: hearing issues, joint damage, urinary incontinence, or dizziness with falls.

**Methods:**

The study sample comprised 25,637 community-dwelling persons aged 65 years and older residing in the Netherlands (Data source: TOPICS-MDS, www.topics-mds.eu). Floor and ceiling effects were examined. To assess convergent validity, random effects meta-correlations (Spearman’s rho) were derived between individual EQ-5D domains and related survey items. To further examine construct validity, the association between sociodemographic characteristics and EQ-5D summary scores were assessed using linear mixed models. Outcomes were compared to the overall study population as well as a ‘healthy’ subgroup reporting no major chronic conditions.

**Results:**

Whereas ceiling effects were observed in the overall study population and the ‘healthy’ subgroup, such was not the case in the geriatric condition subgroups. The majority of hypotheses regarding correlations between survey items and sociodemographic associations were supported. EQ-5D summary scores were lower in respondents who were older, female, widowed/single, lower educated, and living alone. Increasing co-morbidity had a clear negative effect on EQ-5D scores.

**Conclusion:**

This study supported the construct validity of the EQ-5D across four major geriatric conditions. For older persons who are generally healthy, i.e. reporting few to no chronic conditions, the EQ-5D confers poor discriminative ability due to ceiling effects. Although the overall dataset initially suggested poor discriminative ability for the EQ-5D, such was not the case within subgroups presenting with major geriatric conditions.

**Electronic supplementary material:**

The online version of this article (doi:10.1186/s12955-017-0616-x) contains supplementary material, which is available to authorized users.

## Background

Medicine is transitioning away from the traditional single disease model which often dichotomizes health outcomes as merely the presence or absence of disease. Rather, to encompass the far-reaching effects of morbidity, global measures of health, such as health-related quality of life (HRQoL), are now viewed as a critical outcome in contemporary medical research [1]. HRQoL is a subjective, multidimensional concept which incorporates physical, psychological, and social wellbeing. Numerous instruments have been designed to measure HRQoL [2], with a relatively large number targeting condition-specific populations (e.g. measuring HRQoL cancer patients [3]). In contrast, generic HRQoL instruments are intended for use across different (adult) populations, irrespective of underlying conditions. The EQ-5D falls under this latter category and is one of the commonest instruments used to measure generic HRQoL. The EQ-5D was developed by an international task force to permit a brief assessment of an individual’s overall health status across five domains: mobility, self-care, usual activities, pain/discomfort, and anxiety/depression [4]. The EQ-5D can be used to generate a descriptive profile as well as a preference-weighted summary score [4].

Although the broad scope and simplicity of the EQ-5D have made it a widely accessible instrument, these characteristics have also elicited ceiling effects in data collection [5–7]. Thus, a prevailing concern is that the relatively few dimensions may lack the necessary level of responsiveness and discriminative ability to detect health changes for certain health conditions [8]. Numerous studies have therefore investigated measurement properties of the EQ-5D across a wide range of morbidity subgroups [7, 9, 10]. Although construct validity is usually maintained, there have been observed differences in the instrument’s performance. For instance, the EQ-5D was more responsive in persons with urinary incontinence [11] than in persons with hearing impairment [12].

Still, there remains a fundamental need to re-evaluate measurement properties of the EQ-5D in different study populations. Although previous validation work gives credence to the robustness of EQ-5D, the instrument itself is never truly ‘valid’. Rather, validity is a characteristic of a study population [13]; measurement properties are thus intrinsically linked to the target sample (e.g. community-dwelling older persons), the country of the study, how subgroups of interest were identified, and other defining characteristics of the study population. For this reason, preliminary validation of the instrument is necessary prior to in-depth analyses. Moreover, in older persons’ research, there is also a clear benefit to focus on the instrument’s performance across geriatric conditions, i.e. prevalent conditions in older persons which often stem from multifactorial causes [14]. As populations age, chronic geriatric conditions linked to progressive organ failure are of particular interest since they place a growing demand on health care services and further jeopardize health-related quality of life (HRQoL).

The primary aim of this study was to assess the validity and applicability of the EQ-5D in four major chronic geriatric conditions: hearing issues, joint damage, urinary incontinence, or dizziness with falls. These conditions were among the most frequently reported conditions in the dataset under review and are typically prevalent in older populations. Whereas a substantial number of studies have examined singular conditions, we complement previous research by further evaluating the measurement properties of these subgroups against the overall study population as well as a ‘healthy’ subgroup reporting no major chronic conditions. For the purposes of this study, we assessed floor and ceiling effects and construct validity.

## Methods

### Data source

Data were derived from The Older Persons and Informal Caregivers Survey Minimum DataSet (TOPICS-MDS), which is a public access data repository designed to capture essential information on the physical and mental wellbeing of older persons and informal caregivers in the Netherlands. A detailed description of TOPICS-MDS has been presented elsewhere (www.topics-mds.eu) [15]. Briefly, the Dutch National Care for the Elderly Programme was established in 2008 to promote proactive, integrated health care for older persons with complex care needs. As part of this national agenda, TOPICS-MDS was developed to prospectively collect uniform information from all research projects funded under the programme. Thus, TOPICS-MDS consists of pooled data from various research projects which differ across study design, sampling framework, and inclusion criteria. All data were cleaned locally using a standardized protocol. Anonymized individual-level data were then submitted to a central institution (Radboud University Medical Center, Nijmegen, Netherlands) for further validation checks and creation of the pooled dataset. Since various research projects submit information to TOPICS-MDS, the database is dynamic in nature and may be updated with new observations. Our present analysis is based on the second version of the database (available as of May 2015). TOPICS-MDS is a fully anonymized dataset available for public access, and therefore this analysis was exempt from ethical review (Radboud University Medical Centre Ethical Committee review reference number: CMO: 2012/120).

### Population

TOPICS-MDS includes studies which sampled from institutionalized (nursing homes and residential care facilities) and non-institutionalized settings. For the purposes of this study, analyses were based on community-dwelling older persons aged 65 years and older. Older persons residing in nursing homes were excluded due to small numbers, whereas those residing in residential care facilities were excluded since they represent a distinct subgroup of frail older persons. In the community-dwelling population, sampling strategies varied across study protocols; older persons were sampled from primary care centers, hospital settings, or the general population.

### Variables

The EQ-5D assesses five dimensions (mobility, self-care, usual activities, pain/discomfort, anxiety/depression) using a three-level response option (1 = no problems; 2 = some problems; 3 = extreme problems) [16]. When combined, these scores can describe up to 243 (i.e. 3^5^) unique health states, with ‘11111’ and ‘33333’ representing the best and worst possible health states respectively. A summary score can also be derived using a population-specific tariff (weighting); this analysis has applied a tariff validated for the Dutch population [17]. An EQ-5D summary score of one represents the best imaginable health state whereas a score lower than zero represents a health state perceived to be worse than death.

Morbidity status was self-reported, which can be problematic due to under- and over-reporting. However, the study design of TOPICS-MDS did not include clinical evaluation for validation of self-reported data. For the purposes of this study, subgroup analyses focused on the four most prevalent geriatric conditions in the database. Older persons were asked if they had experienced hearing issues, joint damage (defined as arthrosis or degenerative arthritis of the hips or knees), urinary incontinence, or dizziness with falls in the past 12 months. To derive the subgroups, each condition was essentially used as an ‘index condition’, i.e. if a respondent reported the condition they were included in the subgroup. Nonetheless, many respondents reported co-existing index conditions; thus, these four subgroups were not mutually exclusive. The degree of overlap between these subgroups is outlined in Table 1. Thirteen other chronic conditions which are regularly recorded in the older Dutch population were also assessed [18]. Respondents were classified as ‘healthy’ if they did not report any of the 17 conditions collected in TOPICS-MDS. Despite being classified as ‘healthy’, these respondents may have had other conditions not evaluated in TOPICS-MDS, and thus this label must be interpreted with caution in the subsequent analyses.

**Table 1:** Sociodemographic characteristics of community-dwelling older persons aged 65 years and older, TOPICS-MDS, 2015

	Overall sample *N =* 25,637	Healthy^a^ *N =* 2,275	Hearing issues *N =* 9,762	Joint damage^b^ *N =* 11,903	Urinary incontinence *N =* 5,932	Dizziness with falls *N =* 4,273
Age [mean (SD)]	78 (6)	75 (5)	80 (6)	78 (6)	79 (6)	79 (6)
Sex						
Men	41.7	49.2	46.1	29.9	25.0	34.9
Women	58.3	50.8	53.9	70.1	75.0	65.1
Marital status						
Married/cohabiting	53.9	63.7	51.8	48.0	42.9	44.7
Widowed	35.6	26.5	38.8	40.8	45.9	44.0
Single/divorced	10.5	9.8	9.3	11.2	11.2	11.3
Educational level						
Primary	31.7	24.1	33.1	34.5	37.2	37.8
Secondary	49.2	51.6	47.6	48.9	47.5	46.2
College/Some college	19.1	24.3	19.3	16.6	15.4	16.0
Living arrangements						
Lives alone	44.9	34.7	46.8	50.8	54.9	54.0
Lives with others	55.1	65.3	53.2	49.2	45.1	46.0
Morbidity						
Hearing issues	39.3	--	100	42.2	47.0	51.0
Joint damage	48.5	--	52.2	100	61.9	59.8
Urinary incontinence	24.0	--	29.0	31.1	100	37.4
Dizziness with falls	17.3	--	22.5	21.3	26.6	100
Other co-morbidities^c^						
None	21.7	--	15.7	16.2	12.2	10.6
One	31.0	--	28.4	28.6	25.8	23.2
Two	24.0	--	26.1	24.8	25.8	25.6
Three or more	23.4	--	29.8	30.3	36.3	40.7
Requires assistance^d^						
Bathing	15.6	4.1	17.0	19.0	25.4	25.4
Dressing	11.6	3.0	12.3	14.4	18.4	18.3
Walking	25.8	6.8	29.8	34.6	39.9	42.8
IADL score^d^ [median, IQR]	1 (2)	0 (0)	1 (3)	1 (3)	2 (2)	2 (2)
Psychological wellbeing^e^ [mean, SD]	74.2 (18.0)	83.5 (14.0)	72.8 (17.9)	71.3 (18.3)	69.3 (18.7)	65.9 (19.5)
General quality of life^f^ [mean, SD]	7.4 (1.3)	8.0 (1.0)	7.2 (1.3)	7.2 (1.2)	7.0 (1.3)	6.9 (1.4)

Percentages are presented unless other specified

*IADL* Instrumental Activities of Daily Living, *IQR* interquartile range

^a^Reported having none of the 17 chronic conditions recorded in The Older Persons and Informal Caregivers Survey

^b^Defined as arthrosis or degenerative arthritis of the hips and/or knees

^c^Included self-reported diabetes, cerebrovascular events, heart failure, cancer, airway disease, osteoporosis, bone fractures (hip/other), prostrate issues, depression, anxiety, dementia, or vision problems

^d^Modified Katz Index of Independence in Activities of Daily Living. IADL score ranges from zero to eight limitations

^e^Rand mental health subscale (Range 0–100; higher scores represent a more positive emotional state)

^f^Cantril’s Self Anchoring Ladder (Range 0–10; higher scores represent higher perceived quality of life)

There were several variables of interest for validation purposes. Limitations in activity were determined using a modified 15-item Katz Index [19, 20], which included Activities of Daily Living (ADL; i.e. bathing, dressing, toileting, use of incontinence products, transferring, and eating), Instrumental Activities of Daily Living (IADL; i.e. grooming, use of a telephone, travelling, grocery shopping, meal preparation, household tasks, taking medication, and financial management) and an additional indicator for mobility (i.e. walking). Response options were dichotomized as ‘requires assistance’ or ‘does not require assistance’. An IADL summary score was summated, ranging from zero to eight, with higher scores representing greater limitations in activities. Emotional wellbeing was evaluated using the Rand-36 mental health sub-scale [21]. This scale asked how often in the past four weeks an individual has felt: very nervous; calm and peaceful; down-hearted and blue; happy; so down in the dumps nothing could cheer [him/her] up. A five-level response option was presented ranging from ‘never’ to ‘always’. Positive items were scored from zero to 100 whereas negative items were reverse scored. A summary score ranged zero and 100 with higher scores implying a more positive emotional state. Self-perceived general quality of life was assessed with a modified version of Cantril’s Self Anchoring Ladder [22]. Older persons were asked to rate their present life on a scale between zero (completely unsatisfied with life) and ten (completely satisfied with life).

### Statistical analysis

Given that TOPICS-MDS is a pooled dataset, subsequent analyses were derived using a one-step individual patient data meta-analysis [23]. Demographic characteristics of the study population were assessed. Distributional properties were derived for individual EQ-5D items. The mean (standard deviation), range and floor and ceiling effects for the EQ-5D summary score were further derived. To date, there is no general consensus for floor and ceiling effects; thus, these effects were considered to be present if at least 15% of older persons reported either the lowest scores (health state ‘33333’, i.e. weighted score −0.33) or highest scores (health state ‘11111’, i.e. weighted score 1.0) [24].

To assess convergent validity, random effects meta-correlations (Spearman’s rho) were derived using the meta package in R to allow for heterogeneity between individual studies in the pooled dataset [25]. The EQ-5D mobility item was correlated with Katz Index item, ‘assistance with walking’. The EQ-5D self-care item was correlated with two ADL items from the Katz Index, bathing and dressing. The EQ-5D usual activities item was correlated with the summary IADL score. The anxiety/depression item was correlated with the Rand-36 mental health sub-scale summary score. Lastly, the EQ-5D summary score was correlated with a general quality of life score (Cantril’s Self Anchoring Ladder). Correlation coefficients were classified as trivial (≤0.1), weak (0.1 to <0.3), moderate (0.3 to <0.5), strong (0.5 to <0.7), very strong (≥0.7) [26]. A strong to very strong, positive correlation was hypothesized between the EQ-5D mobility, self-care, usual activities items and the Katz walking item, the Katz bathing/dressing items and the Katz IADL summary score respectively. A moderate to strong, negative correlation was anticipated between the EQ-5D anxiety/depression item and the Rand-36 mental health sub-scale summary score. The general quality of life score based on Cantril’s Self Anchoring Ladder is a broad evaluative measure whereas the EQ-5D summary score reflects HRQoL. Given this conceptual distinction, a moderate positive correlation was hypothesized between these scores.

To examine further construct validity and to allow for clustering effects by individual research projects, linear mixed models were performed by regressing the EQ-5D summary score on key demographic variables in the overall study population as well as within subgroups. Variables included age, sex, marital status, educational level, living arrangement (i.e. alone or with others), and morbidity status. For morbidity status, hearing issues, joint damage, urinary incontinence, or dizziness with falls were evaluated individually. Other co-morbidities (i.e. diabetes, cerebrovascular events, heart failure, cancer, airway disease, osteoporosis, fractured hip, other bone fractures, prostrate issues, depression, anxiety, dementia, or vision problems) were collapsed into a single variable and categorized as ‘none’, ‘one co-morbidity’, ‘two co-morbidities’, or ‘ three or more co-morbidities’. HRQoL was hypothesized to be lower in respondents who were older [27], widowed or single (defined as unmarried or divorced) [28, 29], lower educated [27], and living alone [30]. Moreover, women were expected to report lower overall HRQoL [29], and in particular to report higher anxiety and/or depression [27]. Multimorbidity was anticipated to have a strong, negative effect on HRQoL [31, 32]. Associations were examined in unadjusted models as well as models adjusted for age and sex. All statistical analyses were carried out using SPSS (Version 21.0. Armonk, NY, USA: IBM Corp) and R (2013: Vienna, Austria).

## Results

Data were extracted on 25,637 community-dwelling persons aged 65 years and older from 32 research projects. Most were sampled from primary care centers (69.3%, *n =* 17,777), followed by hospital settings (20.6%, *n =* 5,294) and the general population (10.0%, *n =* 2,566). From the overall study population, nearly three-quarters (73.3%, *n =* 18,791) reported at least one of the four geriatric conditions under review; of these respondents, half (50.0%, *n =* 9,400) reported only one geriatric condition whereas the remainder reported two (33.5%, *n =* 6,296), three (13.3%, *n =* 2502), or four (3.2%, *n =* 593) of these conditions. Overall, 8.9% (*n =* 2,275) were classified as healthy, i.e. reported no morbidities.

Sociodemographic characteristics differed across subgroups (Table 1). Respondents comprising the ‘healthy’ subgroup were more likely to be younger, married, higher educated, and reside with others. These respondents reported the highest psychological wellbeing and general quality of life scores. Whereas the average age was broadly similar across geriatric conditions subgroups, there were observable differences in the distributions of sex and marital status. Nearly half of respondents in the hearing issues subgroup were male compared to one-quarter in the urinary incontinence subgroup. Respondents in the urinary incontinence group were the most likely to be widowed. Based on the Katz Index, assistance needed for bathing was disproportionately higher in the urinary incontinence and dizziness with falls subgroups; the median number of reported limitations in IADL was also higher in these subgroups. Respondents in the dizziness with falls subgroup reported the lowest scores for psychological wellbeing and general quality of life.

In the overall study population, there was a clear ceiling effect, with nearly one in five respondents (19.2%) reporting optimal HRQoL (i.e. an EQ-5D score of ‘11111’) (Table 2). This effect was driven, in part, by respondents in the ‘healthy’ subgroup. These respondents were the least likely to report any problems across the five EQ-5D dimensions, and more than half (57.5%) reported optimal HRQoL. Relative to the joint damage, urinary incontinence and dizziness with falls subgroups, respondents in the hearing issues subgroup were the least likely to report any problems across the five dimensions. Yet, despite a higher proportion of respondents in the hearing issues subgroup reporting optimal HRQoL, a ceiling effect was not observed. Irrespective of the subgroup, very few respondents (<1%) reported the worst imaginable health state (i.e. an EQ-5D score of ‘33333’).

**Table 2:** Distributional properties of the EQ-5D summary score and individual dimensions

	Overall sample *N =* 25,637	Healthy^a^ *N =* 2,275	Hearing issues *N =* 9,762	Joint damage^b^ *N =* 11,903	Urinary incontinence *N =* 5,932	Dizziness with falls *N =* 4,273
EQ-5D dimensions						
*Mobility (%)*						
No problems	39.0	77.4	33.1	22.5	22.0	23.2
Slight problems	57.4	21.8	63.8	73.1	72.7	73.1
Extreme problems	3.6	0.8	3.0	4.4	5.2	3.7
*Self-care (%)*						
No problems	79.2	94.9	76.8	73.6	67.2	67.2
Slight problems	15.7	3.8	18.1	20.4	23.6	24.4
Extreme problems	5.1	1.3	5.1	6.0	9.2	8.4
*Usual activities (%)*						
No problems	56.4	86.8	52.3	45.2	41.5	41.4
Slight problems	34.2	10.2	38.4	43.6	45.2	45.0
Extreme problems	9.3	3.0	9.3	11.2	13.3	13.5
*Pain/Discomfort (%)*						
No problems	37.4	71.2	34.3	20.3	25.2	23.6
Slight problems	53.5	27.0	55.7	66.1	60.3	60.1
Extreme problems	9.2	1.8	10.0	13.6	14.5	16.2
*Anxiety/Depression (%)*						
No problems	75.3	91.3	72.7	70.7	65.0	60.3
Slight problems	22.4	8.4	25.0	26.5	30.8	34.3
Extreme problems	2.3	0.3	2.4	2.8	4.2	5.4
EQ-5D summary score						
Mean (SD)	0.72 (0.26)	0.90 (0.16)	0.71 (0.26)	0.65 (0.26)	0.63 (0.28)	0.61 (0.29)
Range	–0.33, 1.00	–0.18, 1.00	–0.33, 1.00	–0.33, 1.00	–0.33, 1.00	–0.33, 1.00
Floor (%)	<1.0	0	<1.0	<1.0	<1.0	<1.0
Ceiling (%)	19.2	57.5	14.9	6.3	7.3	6.8

^a^Reported having none of the 17 chronic conditions recorded in The Older Persons and Informal Caregivers Survey

^b^Defined as arthrosis or degenerative arthritis of the hips and/or knees

Differences in population size and reporting levels across individual dimensions of the EQ-5D attributed to differences in the number of observed health states (defined as the concatenation of domain scores). In the overall study population, 213 out of the 243 potential health states were represented. One hundred ninety-one profiles were represented in the hearing issues subgroup, 194 in the joint damage subgroup, 190 in the urinary incontinence subgroup, and 169 in the dizziness with falls subgroup. In the ‘healthy’ subgroup, 76 different health profiles were observed. Optimal HRQoL was the most frequently reported health state in the overall study population (19.2%) as well as in the ‘healthy’ (57.5%) and hearing issues (14.9%) subgroups (Table 3). In contrast, the most frequently reported profile in the joint damage subgroup was “some issues” with mobility and pain/discomfort and “no issues” with self-care, usual activities or anxiety/depression (13.8%). The urinary incontinence and dizziness with falls subgroups mirrored the joint damage subgroup, with the exception of reporting “some issues” for usual activities (10.5% and 9.4% respectively).

**Table 3:** Ten most frequently reported EQ-5D health states^a^

Overall sample *N =* 25,637 Profile (%)	Healthy^b^ *N =* 2,275 Profile (%)	Hearing issues *N =* 9,762 Profile (%)	Joint damage^c^ *N =* 11,903 Profile (%)	Urinary incontinence *N =* 5,932 Profile (%)	Dizziness with falls *N =* 4,273 Profile (%)
**11111 (19.2)**	**11111 (57.5)**	**11111 (14.9)**	21121 (13.8)	21221 (10.5)	21221 (9.4)
21121 (10.4)	11121 (11.6)	21121 (10.7)	21221 (12.9)	21121 (9.5)	21121 (9.1)
21221 (9.1)	21111 (5.2)	21221 (9.7)	11121 (7.5)	**11111 (7.3)**	**11111 (6.8)**
11121 (8.8)	21121 (5.1)	11121 (7.6)	21222 (6.5)	21222 (6.6)	21222 (6.8)
21111 (5.2)	21221 (2.6)	21111 (5.7)	**11111 (6.3)**	11121 (5.6)	11121 (6.0)
21222 (4.5)	11112 (2.3)	21222 (5.0)	22221 (4.3)	22221 (4.7)	22222 (4.5)
22221 (3.1)	11122 (1.7)	22221 (3.8)	21111 (3.9)	21111 (4.2)	21111 (3.9)
21122 (2.8)	21211 (1.4)	21122 (3.1)	21122 (3.8)	22222 (3.9)	21122 (3.7)
21211 (2.4)	11211 (1.4)	21211 (3.0)	22222 (2.9)	21122 (3.7)	22221 (3.7)
22222 (2.1)	11221 (1.1)	22222 (2.5)	21211 (1.9)	21211 (2.5)	21211 (2.1)

^a^Digits one through five represent respectively: Mobility; Self-care; Usual activities; Pain/Discomfort; Anxiety/Depression; ‘1’ indicates ‘no problems’, ‘2’ indicates ‘some problems’, and ‘3’ indicates ‘extreme problems’ with domain

^b^Reported having none of the 17 chronic conditions recorded in The Older Persons and Informal Caregivers Survey

^c^Defined as arthrosis or degenerative arthritis of the hips and/or knees

The EQ-5D mobility and Katz walking items were moderately correlated in all subgroups (Table 4). The EQ-5D self-care item and Katz bathing and dressing items were very strongly correlated in the ‘healthy’ subgroup and strongly correlated in the geriatric conditions subgroups. A strong correlation was observed between the EQ-5D usual activities item and the Katz IADL summary score in all subgroups except for the dizziness with falls subgroup. In the ‘healthy’ subgroup, a moderate correlation was observed between the EQ-5D anxiety/depression item and psychological well-being (as measured by the Rand-36 mental health subscale score) and a weak correlation was observed between the EQ-5D summary score and a general quality of life score (as measured by Cantril’s Self-anchoring Ladder). This differed from the geriatric condition subgroups which demonstrated strong and moderate correlations for these respective measures.

**Table 4:** Random effects meta-correlation coefficient (Spearman’s rho) between EQ-5D select dimensions/summary score and related survey items

	Overall sample *N =* 25,637 ρ (95% CI)	Healthy^a^ *N =* 2,275 ρ (95% CI)	Hearing issues *N =* 9,762 ρ (95% CI)	Joint damage^b^ *N =* 11,903 ρ (95% CI)	Urinary incontinence *N =* 5,932 ρ (95% CI)	Dizziness with falls *N =* 4,273 ρ (95% CI)
Mobility and Katz mobility item ‘Walking’	0.39 (0.35, 0.43)	0.46 (0.39, 0.53)	0.38 (0.33, 0.42)	0.33 (0.29, 0.37)	0.35 (0.31, 0.39)	0.38 (0.31, 0.44)
Self-care and Katz ADL item ‘Bathing’	0.68 (0.65, 0.71)	0.83 (0.73, 0.89)	0.67 (0.63, 0.70)	0.64 (0.61, 0.68)	0.69 (0.64, 0.73)	0.68 (0.64, 0.71)
Self-care and Katz ADL item ‘Dressing’	0.61 (0.57, 0.64)	0.80 (0.69, 0.88)	0.59 (0.55, 0.62)	0.57 (0.53, 0.61)	0.61 (0.57, 0.64)	0.61 (0.57, 0.65)
Usual Activities and IADL summary score	0.51 (0.47, 0.55)	0.55 (0.41, 0.66)	0.51 (0.47, 0.55)	0.51 (0.46, 0.55)	0.51 (0.46, 0.55)	0.47 (0.43, 0.52)
Anxiety/Depression and Psychological wellbeing^c^	−0.52 (−0.55, −0.49)	−0.36 (−0.42, −0.29)	−0.52 (−0.56, −0.48)	−0.53 (−0.57, −0.50)	−0.55 (−0.59, −0.52)	−0.58 (−0.62, −0.54)
EQ-5D summary score and General quality of life score^d^	0.40 (0.35, 0.45)	0.28 (0.19, 0.37)	0.38 (0.34, 0.43)	0.38 (0.32, 0.43)	0.39 (0.34, 0.43)	0.40 (0.34, 0.47)

*IADL* Instrumental Activities of Daily Living, *ADL* Activities of Daily Living

^a^Reported having none of the 17 chronic conditions recorded in The Older Persons and Informal Caregivers Survey

^b^Defined as arthrosis or degenerative arthritis of the hips and/or knees

^c^Based on the Rand mental health subscale

^d^Based on Cantril’s Self Anchoring Ladder

In the overall study population, the average EQ-5D summary score was higher in respondents who were younger, male, married, more highly educated, and residing with others (Table 5). A clear gradient was observed by co-morbidity status, with fewer co-morbidities resulting in improved HRQoL scores. For instance, compared to a mean EQ-5D summary score of 0.59 (95%CI 0.56, 0.62) among older persons with four or more co-morbidities, older persons with only one co-morbidity had a mean score that was 0.20 (95% CI 0.19, 0.21) higher. When adjusted for age and sex, associations were broadly similar to the unadjusted model. Similar patterns were observed across subgroups in the unadjusted and adjusted models (Additional file 1: Tables S1 and Table S2).

**Table 5:** Unadjusted and adjusted EQ-5D summary scores for the overall population (*N =* 25,637)

	Unadjusted	Adjusted for age and sex^b^
Age		
Mean age^a^	**0.69 (0.66, 0.73)**	--
Per additional year	−0.003 (−0.003, −0.002)	--
Sex		
Men (reference)	**0.73 (0.70, 0.77)**	--
Women	−0.06 (−0.07, −0.06)	--
Marital status		
Married/cohabiting (reference)	**0.71 (0.68, 0.74)**	**0.73 (0.70, 0.77)**
Widowed	−0.03 (−0.04, −0.03)	−0.0001 (−0.01, 0.01)
Single/divorced	−0.03 (−0.04, −0.02)	−0.01 (−0.02, −0.002)
Educational level		
Primary	−0.03 (−0.04, −0.02)	−0.02 (−0.03, −0.02)
Secondary (reference)	**0.70 (0.67, 0.74)**	**0.73 (0.70, 0.76)**
College/Some college	0.03 (0.02, 0.04)	0.02 (0.01, 0.03)
Living arrangements		
Lives alone	−0.03 (−0.03, −0.02)	−0.002 (−0.01, 0.005)
Lives with others (reference)	**0.71 (0.67, 0.74)**	**0.73 (0.70, 0.76)**
Hearing issues		
Yes (reference)	**0.67 (0.65, 0.71)**	**0.72 (0.68, 0.75)**
No	0.03 (0.02, 0.03)	0.02 (0.02, 0.03)
Joint damage^c^		
Yes (reference)	**0.63 (0.60, 0.67)**	**0.66 (0.63, 0.69)**
No	0.12 (0.11, 0.12)	0.11 (0.10, 0.11)
Urinary incontinence		
Yes (reference)	**0.62 (0.58, 0.65)**	**0.66 (0.62, 0.69)**
No	0.10 (0.10, 0.11)	0.09 (0.08, 0.10)
Dizziness with falls		
Yes (reference)	**0.59 (0.56, 0.62)**	**0.63 (0.60, 0.66)**
No	0.13 (0.12, 0.13)	0.12 (0.11, 0.13)
Other co-morbidities^d^		
One co-morbidity	0.20 (0.19, 0.21)	0.19 (0.18, 0.20)
Two co-morbidities	0.15 (0.14, 0.15)	0.15 (0.14, 0.15)
Three co-morbidities	0.10 (0.09, 0.11)	0.10 (0.09, 0.11)
Four or more co-morbidities (reference)	**0.59 (0.56, 0.62)**	**0.63 (0.60, 0.66)**

^a^Mean age was 78 years

^b^References are based on men and the centered mean age for healthy/morbidity subgroups

^c^Defined as arthrosis or degenerative arthritis of the hips and/or knees

^d^Included self-reported diabetes, cerebrovascular events, heart failure, cancer, airway disease, osteoporosis, bone fractures (hip/other), prostrate issues, depression, anxiety, dementia, or vision problems

## Discussion

This study examined the measurement of properties of the EQ-5D across four major geriatric subgroups and supports its validity in the context of TOPICS-MDS. Although the overall study population suggests a ceiling effect in the EQ-5D, this was driven, in part, by a ‘healthy’ subpopulation imbedded within the database. When consideration was given to major geriatric conditions (in essence, as different index conditions) the EQ-5D was found to confer adequate discriminative ability. Reassuringly, the majority of hypotheses regarding correlations between survey items and sociodemographic associations were supported, suggesting that construct validity was maintained. Strong correlations were observed between the EQ-5D self-care item and the Katz Index items for bathing and dressing as well as between the EQ-5D usual activities item and the Katz IADL summary score. Strong correlations were also observed between the EQ-5D anxiety/depression item and the Rand-36 mental health sub-scale summary score. Moderate correlations were observed between the EQ-5D summary and general quality of life scores for the overall study population as well as across morbidity subgroups. Furthermore, EQ-5D scores were lower in respondents who were older, female, widowed/single, lower educated, and living alone. Increasing co-morbidity had a clear negative effect on EQ-5D scores.

However, there were several notable observations regarding the instrument’s performance. Previous research has reported the weak performance of the EQ-5D in older persons with hearing issues [33] and has suggested that the Health Utility Index Mark III (HUI3) possessed better discriminatory ability for HRQoL reporting in this subgroup [33, 34]. Although a borderline ceiling effect was observed in this study, arguably, the EQ-5D still provided an adequate measure of HRQoL in this subgroup. To further test the robustness of the EQ-5D in older persons with hearing issues, future research would ideally assess the responsiveness of the instrument in the context of TOPICS-MDS.

Since the presence of ceiling effects is a well-recognized limitation of the EQ-5D, it was unsurprising that this instrument lacked discriminative ability for ‘healthy’ older persons. However, it is important to emphasize that the database contains relatively few respondents who fall into this category (<10%). Most older persons reported multiple conditions, and for these respondents, the EQ-5D provides a suitable discriminatory ability for HRQoL. Still, in future research studying older populations, it may be prudent to administer the EQ-5D-5L. The EQ-5D-5L was more recently developed to improve discriminatory ability (and thus potentially reducing the risk of ceiling effects) by providing five-level response options across each of the dimensions used in the original EQ-5D.

There were several cases where convergent validity deviated from a priori hypotheses. In all subgroups, only moderate correlations were observed between the EQ-5D mobility item and the Katz walking item. In the dizziness with falls subgroup, there was only a moderate correlation between the EQ-5D usual activities item and the IADL summary score. In the ‘healthy’ subgroup, a weak correlation was observed between the EQ-5D summary score and the general quality of life score. It is uncertain why these findings arose. Speculatively, the Katz walking item may lack sufficient detail for older persons with multimorbidity. Whereas some may argue that that the EQ-5D usual activities domain and the IADL summary score represent conceptually different constructs, it remains unclear why the strength of the correlation would only be affected in the dizziness in falls subgroup. In the healthy subgroup, ceiling effects likely attenuated the correlation between the EQ-5D summary score and general quality of life score (i.e. Cantril’s Self Anchoring Ladder).

Several limitations must be noted. Firstly, data on geriatric conditions were self-reported posing the risk of reporting bias. Underreporting of hearing loss [35] and urinary incontinence [36] in older persons is a well-known phenomenon and may be partly attributed to social embarrassment. Furthermore, there are knowledge gaps in older persons’ understanding of urinary incontinence [37]; involuntary loss of urine during physical exertion or laughing is not always recognized as a form of incontinence. For these reasons, it is likely that these two conditions were underreported in our study. Whereas older persons may not always report dizziness to their health care providers [38], this does not necessarily indicate that this item would be underreported in this survey. Similarly, joint damage is less prone to reporting bias [39]. Nonetheless, in the absence of complementary clinical data, the magnitude of reporting bias for each of these conditions could not be discerned.

Secondly, data on geriatric conditions were only reported in the broadest sense, i.e. presence or absence of the condition. This lack of specificity precluded the examination of known-group validity between respondents with differing severity levels. However, this limitation is not distinct to TOPICS-MDS; many general health surveys are not inherently designed to extract detailed information on specific conditions. Similar to these surveys, TOPICS-MDS was designed to provide a more global perspective of health and wellbeing status. In this regard, it is also important to emphasize that TOPICS-MDS only captures information on 17 chronic conditions common in older populations. A substantial proportion of older persons classified as ‘healthy’ did not report optimal health-related quality of life, and thus, the ‘healthy’ subgroup may have had acute or chronic conditions not captured in this survey. Nonetheless, this subgroup did report higher HRQoL, psychological wellbeing, and general quality of life, suggesting a healthier segment of the population.

Moreover, heterogeneity within TOPICS-MDS is a concern since it is a pooled dataset comprised of research projects with different protocols and sampling frameworks (e.g. samples taken from primary care centers, hospital settings and the general population). To address this issue, meta-analytic techniques were applied. Random effects by research project were included in both the correlation analyses and linear mixed models.

Lastly, in the primary analysis, morbidity subgroups were not mutually exclusive, potentially biasing the interpretation of findings. However, this limitation is not distinct to this dataset, but is rather a widespread issue when conducting morbidity research in older persons. In contemporary medicine, older persons rarely present with a single chronic condition but rather a range of conditions [40]. To address this issue in this study, individual geriatric conditions were regressed on EQ-5D summary scores in the different subgroups to examine the impact of individual conditions as well as co-morbidity on the outcome.

In the arena of quality of life research, this study is highly relevant as it performed a thorough analysis of measurement properties of the EQ-5D across four major geriatric conditions in a large group of older persons and supported the discriminative ability of the EQ-5D. The unique infrastructure of TOPICS-MDS allowed for the pooled analysis of individual patient data from 32 research projects, which in turn granted the opportunity to explore subgroup analyses. Such findings are not only pertinent to users of TOPICS-MDS but also to the broader research community interested in accurate wellbeing measures for use in older populations. Furthermore, this study underlines that TOPICS-MDS, as a data sharing initiative, collects key variables for assessing quality of life and wellbeing in older persons.

## Conclusion

This study supported the construct validity of the EQ-5D in the overall TOPICS-MDS study population as well as across older persons presenting with four major geriatric conditions: hearing issues, joint damage, urinary incontinence, and dizziness with falls. Relative to the other three conditions, the risk of ceiling effects was higher for persons with hearing issues. For older persons who are generally healthy, i.e. reporting few to no chronic conditions, the EQ-5D confers poor discriminative ability. Although the pooled dataset for TOPICS-MDS may initially suggest poor discriminative ability for the EQ-5D, such is not the case when a healthy subgroup is distinguished from subgroups presenting with major geriatric conditions.

## Additional file

Additional file 1:
**Table S1.** Unadjusted EQ-5D summary scores by geriatric condition. **Table S2.** Age and sex adjusted EQ-5D summary scores by sociodemographic characteristics. (DOC 111 kb)
