# Supplementary material for: Measurement properties of the EQ-5D across four major geriatric conditions: Findings from TOPICS-MDS
Source: Health Qual Life Outcomes. 2017 Mar 3;15:45. doi: 10.1186/s12955-017-0616-x (PMC5335758; doi:10.1186/s12955-017-0616-x)
Supplement: Additional file 1: — Table S1. Unadjusted EQ-5D summary scores by geriatric condition. Table S2. Age and sex adjusted EQ-5D summary scores by sociodemographic characteristics. (DOC 111 kb) [file 12955_2017_616_MOESM1_ESM.doc]

January 20, 2017

Dear Dr. Claudio Bilotta:

We would like to thank you and the reviewers for your informative and positive critique of our manuscript entitled, “**Measurement properties of the EQ-5D across four major geriatric conditions: Findings from TOPICS-MDS**”, and for the opportunity to resubmit our manuscript to *Health and Quality of Life Outcomes*. Please find attached our revised manuscript; all authors of the original manuscript have approved the revised version.

You will find our responses to the reviewers’ minor queries below; revised text is displayed in blue font in the manuscript. We hope that the revised manuscript now satisfactorily meets the expectations of the editorial staff and reviewers, though we encourage further discourse if any concerns remain.

We appreciate your continued consideration of this manuscript. Please direct any correspondence to Dr. Jennifer Lutomski.

Sincerely yours, on behalf of all co-authors,

Jennifer E. Lutomski

Department of Geriatric Medicine

Radboud University Medical Center

Postbus 9101

6500 HB Nijmegen

E-mail: jennifer.lutomski[@radboudumc.nl](mailto:m.laros@ger.umcn.nl)

Tel: +31-24-361-4868; Fax: +31-24-361-7408

**REVIEWER 1**

**Comment 1.1:** *Well done. Your study's methods are robust, and it is very well written. You have addressed all of the obvious limitations in your discussion.*

**Response 1.1:** We thank the Reviewer for this positive evaluation of our manuscript.

**REVIEWER 2**

*This is a generally well conducted study in an important area. I have a few minor comments which the authors need to address to improve clarity and understanding for the reader.*

**Comment 2.1:** *Introduction: 'cancer patients have their own HrQoL instruments' I suggest to re-phrase to emphasize what is meant here in that here are a relatively large number of condition specific HrQoL instruments. However EQ-5D represents a generic instrument which in principle means that it can be applied across all (adult) populations and conditions.*

**Response 2.1:** The text has been updated on Page 3, Lines 33-37 in line with this recommendation.

Numerous instruments have been designed to measure HRQoL [2], with a relatively large number targeting condition-specific populations (e.g. measuring HRQoL cancer patients [3]). In contrast, generic HRQoL instruments are intended for use across different (adult) populations, irrespective of underlying conditions. The EQ-5D falls under this latter category and is one of the commonest instruments used to measure generic HRQoL.

**Comment 2.2:** *Introduction: I recommend changing the term 'quick assessment to 'brief assessment' to offer a more accurate indication as the term quick may be interpreted negatively to imply an ill-considered or rushed assessment.*

**Response 2.2:** The word ‘quick’ has been replaced with ‘brief’ on Page 3, Line 38.

**Comment 2.3:** *EQ-5D-3L lacks responsiveness and discriminative ability but surprisingly there is no mention in this manuscript(unless I have missed it) of the new 5 level version which is designed to overcome these types of criticisms? This issue needs raising as it is important.*

**Response 2.3:** The added benefits of the EQ-5D-5L is now described on Page 13, Line 268-271.

Still, in future research studying older populations, it may be prudent to administer the EQ-5D-5L. The EQ-5D-5L was more recently developed to improve discriminatory ability (and thus potentially reducing the risk of ceiling effects) by providing five-level response options across each of the dimensions used in the original EQ-5D.

**Comment 2.4:** *Limitations of self-reported conditions needs highlighting from the outset. Also not reporting any of the 17 conditions collected in TOPICS-MDS does not necessarily equate to healthy. Whilst this issue is raised in the Discussion this point also needs acknowledgement from the outset.*

**Response 2.4:** The risks of self-reported data are now emphasized in the Methods section to forewarn readers of potential biases (Page 6, Lines 108-110 and Page 6 Lines 119-121).

Morbidity status was self-reported, which can be problematic due to under- and over-reporting. However, the study design of TOPICS-MDS did not include clinical evaluation for validation of self-reported data.

Despite being classified as ‘healthy’, these respondents may have had other conditions not evaluated in TOPICS-MDS, and thus this label must be interpreted with caution in the subsequent analyses.

**Comment 2.5:** *no explanation or justification is given as to why individuals from institutionalized settings were excluded from the data analysis? With the ageing of populations internationally such individuals are making up greater proportions of the total sample of older people (especially for the oldest old - currently the fastest growing sub-group in many countries). What are the implications of excluding these people and why were the excluded specifically?*

**Response 2.5:** A priori reasoning for excluding institutionalized settings is now clarified in the Methods section (Page 5, Lines 94-96).

Older persons residing in nursing homes were excluded due to small numbers, whereas those residing in residential care facilities were excluded since they represent a distinct subgroup of frail older persons.

**Comment 2.6:** *It is not clear to me how the inevitable overlaps between the four main categories and other co-morbidities were handled in the data analysis. It appears from the tables presented that individuals were largely categorized on the basis of the self-reporting of a single condition? However presumably at least a proportion of individuals may have indicated the presence of two three or all four of the identified conditions? What proportions of individuals reported the presence of more than one of the four conditions and how was this handled in the data analysis?*

**Response 2.6:** The derivation of subgroups is now described in greater detail (Page 6, Lines 113-117). We further describe in the Statistical Analysis subsection how main conditions were treated in comparison with other co-morbidities (please refer to response 2.7).

To derive the subgroups, each condition was essentially used as an ‘index condition’, i.e. if a respondent reported the condition they were included in the subgroup. Nonetheless, many respondents reported co-existing index conditions; thus, these four subgroups were not mutually exclusive. The degree of overlap between these subgroups is outlined in Table 1.

The overlap between the four conditions in each of the subgroups is emphasized in Table 1 as well as in the Results section (Page 9, Lines 183-187).

From the overall study population, nearly three-quarters (73.3%, n=18,791) reported at least one of the four geriatric conditions under review; of these respondents, half (50.0%, n=9,400) reported only one geriatric condition whereas the remainder reported two (33.5%, n=6,296), three (13.3%, n=2502), or four (3.2%, n=593) of these conditions.

**Comment 2.7:** *A related point it is not clear how the 'other comorbidities' category has been developed and whether this includes any of the four main conditions or not? I am also not clear as to how the values presented in Table 5 and in the supplementary tables for the presence of four or more co-morbidities should be interpreted. These values appear implausibly high to me (whilst in contrast the values assigned for the presence of one two or three co-morbidities are really very low) which makes me question the reliability of the data source in this regard? More explanation is needed for these findings which at first sight appear somewhat contradictory.*

**Response 2.7:** We now describe in the Statistical Analysis subsection how the main conditions were treated in comparison with other co-morbidities (Page 8, Lines 166-170).

For morbidity status, hearing issues, joint damage, urinary incontinence, or dizziness with falls were evaluated individually; other co-morbidities (i.e. diabetes, cerebrovascular events, heart failure, cancer, airway disease, osteoporosis, fractured hip, other bone fractures, prostrate issues, depression, anxiety, dementia, or vision problems) were collapsed into a single variable and categorized as ‘none’, ‘one co-morbidity’, ‘two co-morbidities’, or ‘ three or more co-morbidities’.

In Table 5, the four or more co-morbidity category is the reference group; scores are markedly higher for those with fewer co-morbidities. This is in line with previous research and does support the validity of the database. However, this may be misinterpreted if wrong reference group is presumed. To facilitate interpretation of this variable (and supplementary tables), we now provide an example in the main text (Page 10, Lines 233-237).

A clear gradient was observed by co-morbidity status, with fewer co-morbidities resulting in improved HRQoL scores. For instance, compared to a mean EQ-5D summary score of 0.59 (95%CI 0.56, 0.62) among older persons with four or more co-morbidities, older persons with only one co-morbidity had a mean score that was 0.20 (95% CI 0.19, 0.21) higher.
